# Optimization of Nanohybrid Biosensors Based on Electro-Crosslinked Tannic Acid Capped Nanoparticles/Enzyme

**DOI:** 10.3390/molecules27103309

**Published:** 2022-05-21

**Authors:** Rémy Savin, Christian Blanck, Nour-Ouda Benzaamia, Fouzia Boulmedais

**Affiliations:** 1 Institut Charles Sadron, University of Strasbourg CNRS, UPR 22, 67034 Strasbourg, France; savin.remy@gmail.com (R.S.); christian.blanck@ics-cnrs.unistra.fr (C.B.); nour.benzaamia@gmail.com (N.-O.B.); 2 International Center for Frontier Research in Chemistry, 67083 Strasbourg, France

**Keywords:** bioconjugates, glucose oxidase, peroxidase, electrodeposition, electropolymerization, catechol, gallol, nanoarchitectonics, biosensors

## Abstract

Enzymes/Nanoparticles (NPs) bioconjugates are massively used nowadays to develop thin films for optical and electrochemical biosensors. Nevertheless, their full characterization as a thin coating onto electrodes remains little discussed, in particular the influence of NPs size and enzyme/NPs ratio used in the electrodeposition solution. In this study, GOx (160 kDa) and HRP (44 kDa) were used in association with tannic acid capped gold NPs (a series with sizes from 7 to 40 nm) to electrodeposit biosensor coatings, sensitive towards glucose and H_2_O_2_, respectively. The electrodeposition process was based on a mussel-inspired electro-crosslinking between gallol moieties of tannic acid (at the surface of NPs) and amine moieties of the enzymes. On one hand, the sensitivity of the GOx/NPs coatings depends strongly on the NP size and the enzyme/NPs molar ratio of the electrodeposition solution. An optimal sensitivity was obtained by electrodeposition of 11 nm NPs at a GOx/NPs molar ratio close to the theoretical value of the enzyme monolayer. On the other hand, a modest influence of the NPs size was found on the sensitivity in the case of the electrodeposited HRP/NPs coatings, reaching a plateau at the HRP/NPs molar ratio close to the value of the theoretical enzyme monolayer. In both cases, the enzyme/NPs molar ratio played a role in the sensitivity. To fully understand the parameters driving the biosensor sensitivity, a comprehensive evaluation of the colloidal state of the bioconjugates is proposed here.

## 1. Introduction

Nanoparticle/enzyme bioconjugates have received great attention in the development of optical [1,2] or electrochemical [3,4] biosensors. The purpose is to combine the biological activity of the enzyme and its specificity towards an analyte with the unique properties of the nanoparticles (NPs) such as plasmonic band, surface-to-volume ratio, and/or conductivity to translate the biological signal of recognition into a quantifiable and processable one. Several studies showed that the curvature of NPs affects strongly the conformation of the adsorbed protein [5,6,7,8,9,10,11]. However, contradictory trends were found regarding the activity of the adsorbed enzyme. For instance, Tadepalli et al. observed a significant decrease in the adsorbed horseradish peroxidase (HRP) activity with the increase of the gold NPs diameter from 10 to 40 nm [11], and Wu et al. the opposite trend with a similar size range (5–60 nm) [9]. Both studies used two different co-substrates which can explain this inconsistency since the conformation change may affect differently the co-substrate/enzyme interaction. Another difference can be pointed out as in the first study the equivalent surface area of NPs was kept constant by adjusting the optical density value [11] whereas in the second one the NPs concentration (optical density) was kept constant [9]. Breger et al. studied the influence of the gold NP size (1.5–100 nm) using phosphotriesterase keeping constant the enzyme concentration and the surface density of NPs, varying the NPs concentration. In this case, the highest enzymatic activity was obtained with an optimum gold NPs size of 10 nm [12]. Two antagonist effects are often competing: (i) large NPs adsorb more enzymes than small ones leading to higher activity per NPs [13,14], and (ii) small NPs seem to impact the activity of the adsorbed enzymes less than the large ones [12,15,16]. Thus, an optimal NP size is eventually found at a given enzyme and NPs concentrations. Moreover, the protein size regarding NP size was recently found to be also crucial [13]. As a general trend, the activity of large enzymes (>100 kDa) increases with the NPs size [15,16,17] whereas the activity of small enzymes (<50 kDa) decreases with the increase of NPs size [11,18,19]. To the best of our knowledge, there is no exhaustive study on the effect of NPs size on the sensitivity of enzyme/NPs thin coatings. Most of the biosensor studies investigating enzymes/NPs coatings usually use small NPs to acquire a high electroactive surface with very concentrated enzyme aliquots (~20–200 µM) to achieve the highest sensitivity [20]. Until now, there is no evidence that the trends observed in solution will be similar in the case of thin films.

**Figure 1:**
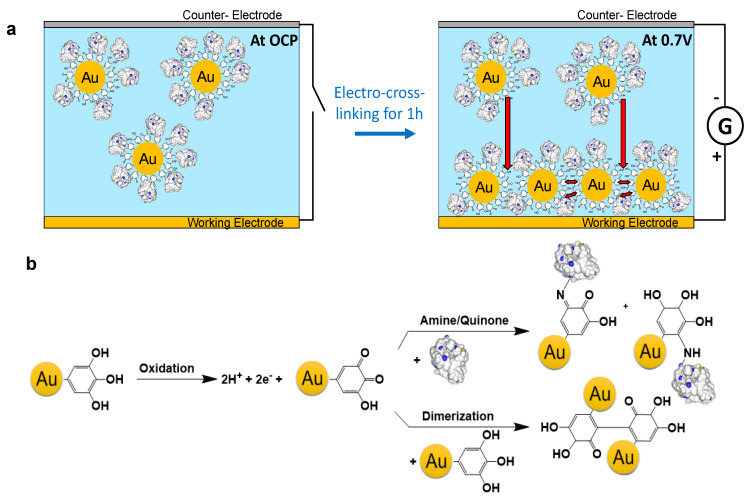
(**a**) Schematic representation of the electro-crosslinking of TA capped gold NPs and enzymes onto an electrode by application of an anodic potential. (**b**) Mechanism of the electro-cross-linking through electro-oxidation of gallol moieties of TA, present at the surface of NPs, into quinone. This oxidation is followed by the chemical reaction of quinone moieties with free amino moieties of the enzyme leading to imine and Michael adduct and with gallol moieties of TA (dimerization).

In this work, we studied the influence of the size of gold NPs and the enzyme/NPs molar ratio of the electrodeposition solution on the sensitivity of the nanohybrid biosensor. For this purpose, we used two glycosylated enzymes having two different sizes HRP (44 kDa, globular size 5.5 nm [21]) and glucose oxidase (GOx) (160 kDa, globular size 6.9 nm [22]) (Table S1 in the Supplementary Materials) and a series of tannic acid (TA) capped gold NPs ranging from 7 to 40 nm. The nanohybrid biosensors were obtained by a one-step mussel-inspired electro-cross-linking process (Figure 1a) using an electrodeposition solution of enzyme/NPs and ferrocene methanol (FcOH), acting as a mediator during the electrodeposition [23]. The presence of gallol moieties on the surface of NPs allowed the adsorption of the enzyme, through hydrogen bonds and hydrophobic interactions. During the application of the anodic potential, FcOH molecules are electro-oxidized into ferrocenium, able to oxidize gallol moieties of TA into quinone. The immobilization of enzyme/NPs is thus obtained by a covalent reaction of quinone with amine moieties of the enzyme and gallol moieties of NPs (Figure 1b). The obtained nanohybrid coating can then be used for the electrochemical detection of the analyte (glucose or H_2_O_2_) using FcOH as a free mediator in solution [23]. This study aims to provide some general thoughts on the parameter driving the sensitivity of enzyme/NPs biosensors made by electrodeposition methods.

## 2. Results and Discussion

### 2.1. Gold Nanoparticle Synthesis and Characterization

**Figure 2:**
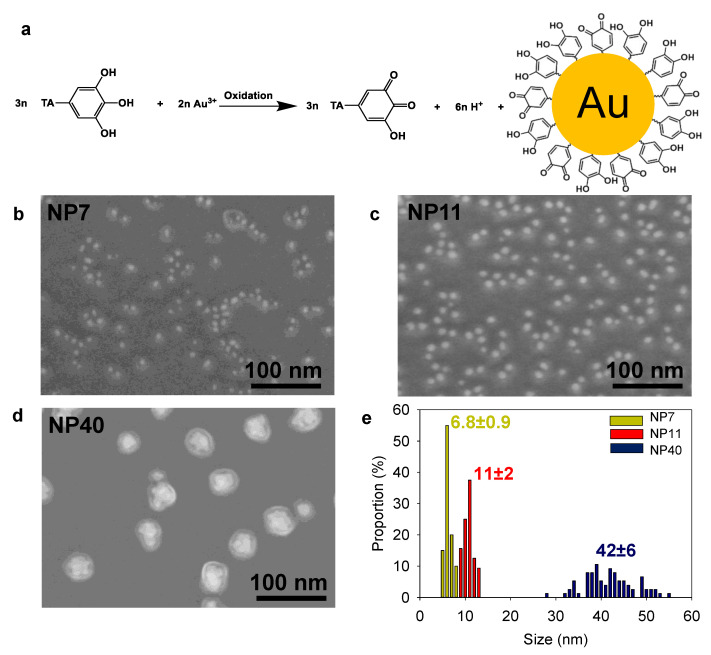
(**a**) Redox synthesis of tannic acid capped gold nanoparticles (NPs) based on TA and gold salt spontaneous reaction. SEM images of (**b**) NP7, (**c**) NP11, (**d**) NP40 and (**e**) their size (in nm) distribution retrieved from TEM size analysis.

NPs were prepared using an adapted protocol from Sivaraman et al. [24] based on the chemical reduction of gold salts by tannic acid (Figure 2a). The reaction proceeds in two stages: the production of “seeds” and their growth until the gold salt is consumed [24,25]. To obtain different diameters, the reactivity of the gold salt solution was adjusted by controlling the pH with the addition of K_2_CO_3_. The increase of the pH induces a change of the dominant species from [AuCl_4_]^−^ to [AuCl_3_(OH)]^−^, [AuCl_2_(OH)_2_]^−^, [AuCl(OH)_3_]^−^ or [Au(OH)_4_]^−^ species [26]. By adjustment of the pH, NPs with a diameter of 11 ± 2 and 42 ± 6 nm were obtained and denoted NP11 and NP40, respectively.

To further decrease the NPs size, ferrocene boronate was added to the tannic acid solution as a fast reducing agent (higher seed density) [27], leading to NPs with a diameter of 6.8 ± 0.9 nm, named NP7. All NPs suspensions appeared in a non-aggregated form (Figure 2b–e). NP40 had a larger standard deviation as illustrated by its polydispersity. The size analysis by dynamic light scattering is given in Figure S1 in the Supplementary Materials. After their synthesis, the concentration of the NPs suspensions was determined (Table S2 in the Supplementary Materials).

### 2.2. Gold Nanoparticles Electrodeposited Coatings

**Figure 3:**
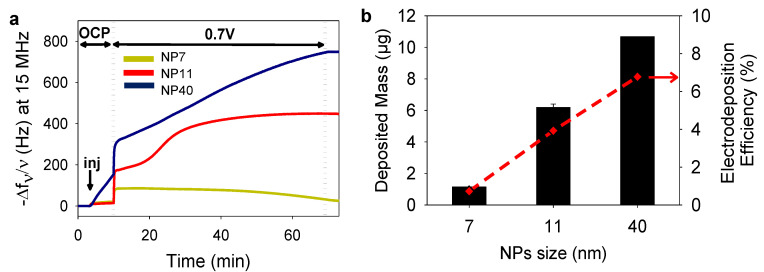
Electrodeposition of NPs with different sizes (**a**) Evolution of the normalized frequency shift of NPs/FcOH solutions, measured by EC–QCM, with NP7, NP11, and NP40, as a function of time during the application of 0.7 V. No electrical stimulus is applied during the OCP (open circuit potential). The arrow “inj” indicates the time of injection of the solution. (**b**) Deposited mass (µg), calculated from EC-QCM, using a geometric surface of 0.8 cm^2^, and the corresponding electrodeposition efficiency (red diamond) for the different NP sizes.

After the synthesis of the NPs, we first studied the electro-crosslinking of NPs based on the dimerization process (Figure 1b). A gold crystal was used as the working electrode and the electroactive surface area (EASA) was calculated depending on the NPs size, keeping the concentration in gold constant in the electrodeposition solution. To this aim, the NPs suspensions were centrifuged and concentrated ten times by addition of 5 mM FcOH/50 mM NaCl aqueous solution. The suspensions were stable in 50 mM NaCl with an average zeta potential of −60 ± 20 mV. The salt acts as a supporting electrolyte and FcOH as a catalytic mediator for tannic acid oxidation. Electrochemical quartz crystal microbalance (EC–QCM) was used to monitor in situ the evolution of the normalized frequency shift, proportional in a first approximation to the adsorbed mass. At the injection of the FcOH/NPs mixture, an increase in the normalized frequency shift was observed originating from the physisorption of NPs (Figure 3a). After stabilization of the signal, the application of 0.7 V induced an instantaneous increase of the frequency shift followed by a slower phase. After 1 h, the coating was rinsed with 50 mM NaCl solution leading to a small decrease in the signal. Since the deposited coating is rigid with $\left(\frac{\Delta D\times f_{0}}{\Delta f}<1\right)$, the Sauerbrey equation was applied to calculate the deposited mass [28]. The increase of the NPs size leads to the increase of the deposited mass (Figure 3b). The efficiency of the electrodeposition was then determined by the ratio between the number of NPs deposited and present in the electrodeposition solution in contact with the electrode (Figure 3b). The number of deposited NPs was estimated using the mass of a single NP, the deposited mass and the geometric surface of the electrode (0.8 cm^2^ with a roughness <1 nm [29]). The overall number of NPs of the electrodeposition solution in contact with the electrode (between the cathode and the anode) can also be roughly estimated by knowing the internal cell volume (100 µL) [29]. The efficiency of the electro-cross-linking was 0.7%, 3.9%, and 6.8% for NP7, NP11, and NP40, respectively. In comparison, an efficiency of ~7% is reached for the electrophoretic deposition of NP (size from 5 to 8 nm) in toluene at 100 V for 1 h [30]. Using a similar size of NPs, the electro-cross-linking process is less efficient than EPD [31] but with two main advantages: it is performed in mild conditions (aqueous NaCl 50 mM, pH 7, 0.7 V) and allows the formation of covalent bonds leading to more robust coatings.

**Figure 4:**
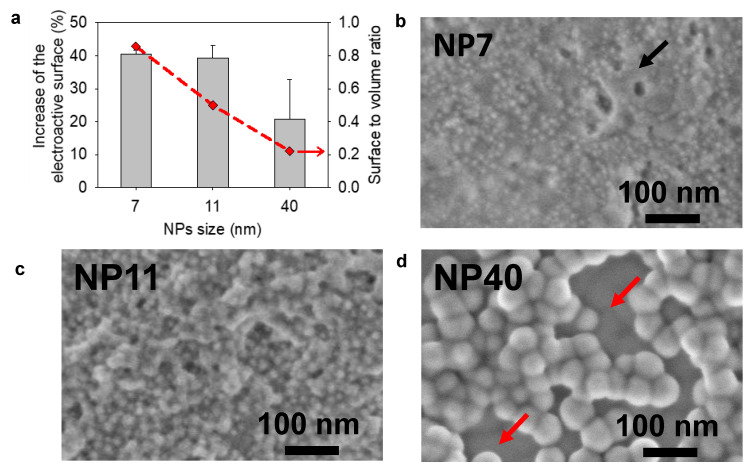
Electrodeposition of NPs with different sizes: (**a**) relative increase of the electroactive surface area (EASA) and surface to volume ratio of NPs (red diamond) as a function of NP size; SEM micrographs of the obtained coatings using (**b**) NP7, (**c**) NP11 and (**d**) NP40. Red arrows indicate the presence of an uncoated area where the underlying electrode is visible and the black arrow the presence of an organic area.

The EASA of the electrode was determined before and after the electrodeposition of NPs as it is involved in the electron exchange of freely diffusing molecules (Figure 4a). In agreement with the surface to volume ratio of NPs, an increase of 40% of the EASA is obtained after the electrodeposition of NP7 and NP11 and only 20% with lower reproducibility with the electrodeposited NP40. SEM micrographs of the electrodeposited NPs coatings showed a full and homogeneous coverage of the electrode for NP7 and NP11 with few organic areas (Figure 4b). On the contrary, NP40 gave heterogeneous coatings with the presence of small aggregates, probably linked to their flocculation [32]. This could explain the low reproducibility of these coatings. For comparison, the SEM image of a bare gold QCM crystal, used as a working electrode, is shown in Figure S2 in the Supplementary Materials.

### 2.3. Sensitivity Optimization of Enzyme/NPs Electrodeposited Coatings

After the study of electrodeposited NPs coatings, the electrodeposition of enzyme/NPs was optimized in terms of sensitivity using two enzymes of different sizes, Horseradish peroxidase (HRP) and glucose oxidase (GOx). The biosensor sensitivity is one of the most important parameters. It corresponds to the proportionality coefficient between the output current value collected at the electrode and the initial substrate concentration in solution. The higher the sensitivity, the better the resolution of the biosensor (i.e., its ability to separate close concentrations). In this study, the sensitivities of GOx/NPs and HRP/NPs based coatings were measured by chronoamperometry with successive addition of their substrates (at 0.25 V with glucose and at 0.19 V with H_2_O_2_, respectively) in the presence of FcOH as mediator. This electrocatalytic signals of GOx/NPs towards glucose and HRP/NPs towards H_2_O_2_ were proven by cyclic voltammetry (Figure S4a,b in the Supplementary Materials). In the case of GOx/NPs, a total disappearance of the ferrocene reduction peak at 0.19 V and the overexpression of the oxidation peak at 0.25 V were observed by increasing the concentration in glucose. In the case of HRP/NPs, a total disappearance of the ferrocene oxidation peak at 0.25 V and the overexpression of the reduction peak at 0.19 V were observed increasing the concentration in H_2_O_2_.

**Figure 5:**
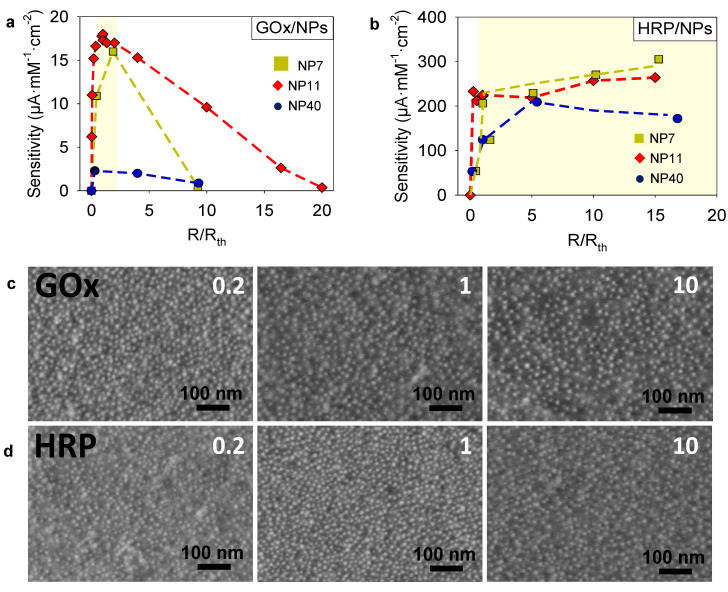
Characterization of GOx/NPs and HRP/NPs coatings. Evolution of the sensitivity of: (**a**) GOx/NPs and (**b**) HRP/NPs coatings with NP7, NP11, and NP40 as a function of the mixture ratio, *R*/*R*_th_, with *R*, the enzyme/NPs ratio of the electrodeposition solution and *R*_th_, the theoretical enzyme/NPs molar ratio to obtain an enzyme monolayer at the surface of NPs (dashed line as a guide to the eye). SEM micrographs, with secondary electrons detector of (**c**) GOx/NP11 and (**d**) HRP/NP11 coatings obtained at different enzyme/NP ratios, the number of each corresponds to the *R*/*R*_th_ value.

The electrodeposition of enzyme/NPs was performed in the presence of 5 mM FcOH/50 mM NaCl for 1 h by applying a potential of 0.7 V (vs. Ag/AgCl). The choice of electrodeposition time was made according to a preliminary study on the GOx/AuNPs system by EC–QCM (Figure S4c in the Supplementary Materials). The mass adsorbed and the sensitivity of GOx/AuNPs were smaller with shorter electrodeposition times (30 min). At longer duration (2 h), the mass adsorbed increased but lower sensitivity was observed. The bioconjugates layers too far from the electrode can no longer communicate with the working electrode. These latter active layers rather tend to limit the diffusion of the mediator and the enzyme substrate in the film which lead to a decrease of the overall film sensitivity. The electro-cross-linking of GOx/NPs and HRP/NPs solutions were studied using the series of synthesized NPs, keeping the gold concentration constant, and varying the enzyme/NP molar ratio for each NPs size. To this aim, the NPs suspensions were centrifuged and concentrated ten times by addition of the adequate amount of enzyme (GOx or HRP) prepared in 5 mM FcOH/50 mM NaCl. To be able to compare the deposited mass and sensitivity of the different coatings, the mixture ratio *R*/*R*_th_ was introduced with *R*, the enzyme/NPs ratio of the electrodeposition solution, and *R*_th_, the theoretical enzyme/NPs molar ratio required to obtain an enzyme monolayer at the surface of NPs. *R*_th_ was defined as the number of small non-deformable enzyme spheres that can be stacked around a single nanoparticle (Section 1 and Figure S3 in the Supplementary Materials). Afterward, the sensitivity of the coatings was determined by chronoamperometry, i.e., by application of 0.25 V for GOx/NPs coatings and 0.19 V for HRP/NPs coatings, and measuring the current density, at different concentrations in glucose of H_2_O_2_ in the presence of FcOH. Ferrocene methanol is therefore not only used to initiate electroreticulation but also to enable the measurement of the electrocatalytic activity of the immobilized enzymes. Then, the coating sensitivity was plotted as a function of the ratio *R*/*R*_th_ (Figure 5a). Raw sensitivity plots are given in the Supplementary Material (Figure S5a,b in the Supplementary Materials). For all tested NPs sizes, the optimal values in sensitivity were obtained at the mixture ratios *R*/*R*_th_ close to 1, where *R*, the value of the enzyme/NPs molar ratio, is close to the theoretical monolayer. The adsorbed mass followed the same behavior with optimal values at mixture ratios (Figure S6a in the Supplementary Materials). At low GOx/NPs molar ratio (*R* < *R*_th_), uncoated and poorly coated NPs were electrodeposited leading to a low quantity of deposited enzyme and thus low enzymatic activity. The drastic decrease in sensitivity observed for a high GOx/NPs molar ratio (*R* > *R*_th_) was concomitant to the decrease in mass adsorbed. The dense corona of enzymes onto the NPs prevents probably the electro-crosslinking process involving gallol moieties of TA. For a given mixture ratio, the sensitivity of the coatings also presents an optimum value depending on the NP size with the following decreasing order NP11 > NP7 > NP40. The highest sensitivity, 17.5 ± 0.4 µA·mM^−1^·cm^−2^, is obtained for the GOx/NP11 coating at a mixture ratio of 27 (*R*/*R*_th_ = 0.91) and with an adsorbed mass of 10 µg obtained from the Sauerbrey equation.

A similar study was carried out for HRP/NPs coatings where the sensitivity toward H_2_O_2_ was evaluated by chronoamperometry at different concentrations in the presence of FcOH (Figure 5b). Different controls were carried out to validate that electrocatalytic signal observed in chronoamperometry at 0.19 V comes from the activity of HRP. The sensitivity of NP11 film towards different H_2_O_2_ concentrations was studied and no electrocatalytic signal was obtained at 0.19 V (Figure S7a in the Supplementary Materials). Indeed, the electro reduction of H_2_O_2_ at the electrode is observed at a lower potential such as –0.5 V. Contrary to the previous system, the sensitivity of HRP/NP films increased linearly with the *R*/*R*_th_ mixture ratio until a value close to 1 followed by a slower increase for small NPs (NP7 and NP11) or a slight decrease for NP40 (Figure 5b). The adsorbed mass follows the same trend reaching almost a plateau at the *R*/*R*_th_ value between 1 and 2 (Figure S6b in the Supplementary Materials). Thus, keeping the NPs concentration constant (Table S1 in the Supplementary Materials), the increase in HRP concentration in the electrodeposition solution did not impact the electro-crosslinking process as observed for GOx. Even in the presence of a large excess of HRP, gallol moieties sites on NPs seem to be accessible to react covalently, after oxidation, with other NPs or enzymes present in the solution. It is likely that for small NPs free HRP are also physically incorporated in the coating leading to the small increase in sensitivity for high values of *R*/*R*_th_ (NP7 and NP11). Similar sensitivities were obtained independently of the NP size at the optimal ratio (*R*/*R*_th_ ~ 1). The highest sensitivity, 305 µA·mM^−1^·cm^−2^, was obtained for HRP/NP7 coating at *R*/*R*_th_ ~ 15. Interestingly, an increase in the linear range was observed for HRP/NPs system when the *R*/*R*_th_ mixture ratio was increased shifting the highest detectable H_2_O_2_ concentration from 100 µM at *R*/*R*_th_ ~ 0.2 to 500 µM at *R*/*R*_th_ ~ 10 (Figure S8a in the Supplementary Materials). These results suggest a higher amount of active enzyme included in the HRP/NPs coating when the concentration in the enzyme is increased. Moreover, the sensitivity plot at the highest tested ratio (R/R_th_ ~10) shows that FcOH concentration significantly modifies the linear range (Figure S8b in the Supplementary Materials). FcOH diffusion is probably the limiting kinetic of the overall system in the tested range for FcOH and H_2_O_2_ concentrations. It suggests that the HRP catalytic reaction kinetic is very fast compared to the FcOH mediated electron transfer. Figure 5c,d depicts the SEM micrographs of GOx/NP11 and HRP/NP11 coatings obtained with increasing the *R*/*R*_th_ mixture ratio showing an increase in organic materials content due to the enzymes. It can be concluded that fine-tuning both NPs size and enzyme/NP molar ratio is required to achieve an optimal sensitivity.

### 2.4. Enzyme/NPs Bioconjugate Characterization

**Figure 6:**
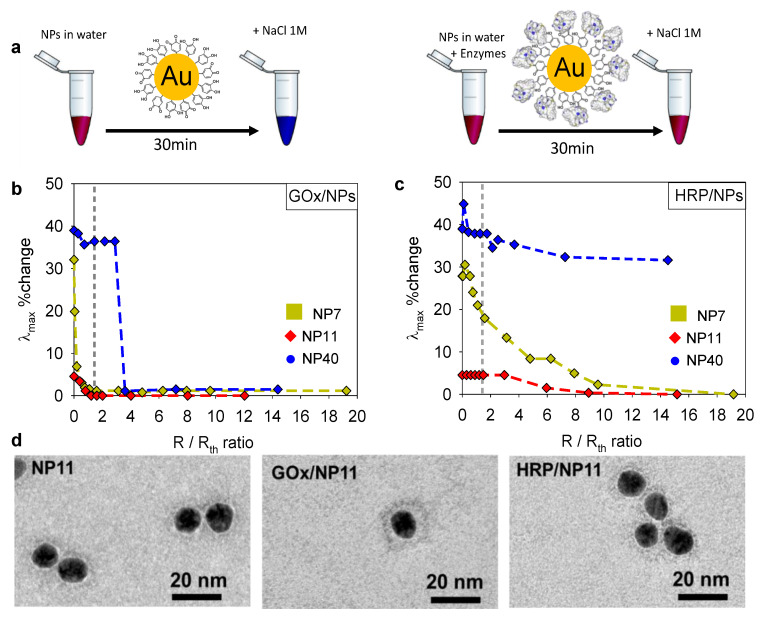
(**a**) Illustration of the gold NPs flocculation assay performed at 1 M NaCl in the absence and the presence of the enzyme. (**b**,**c**) Evolution of the relative change in λ_max_ (i.e., the shift in wavelength/λ_max_(NP)) as a function of the mixture ratio, *R*/*R*_th_ of (**b**) the GOx/NPs suspensions, and (**c**) the HRP/NPs suspensions with NP7, NP11, and NP40. (**d**) TEM images of NP11, GOx/NP11 and HRP/NP11 bioconjugates, both obtained at *R*/*R*_th_ = 10 after uranyl acetate negative staining.

To acquire further insight into the differences found between GOx/NPs and HRP/NPs coatings, we studied the colloidal state of the enzyme/NPs bioconjugates using NP7, NP11, and NP40. It is known that full coverage of NPs by proteins prevents their aggregation in the presence of salt. To determine the optimal enzyme/NPs molar ratio for full coverage of the NPs, a flocculation assay was performed at different molar ratios with the addition of 1 M NaCl [22]. The aggregation of gold NPs leads to a color change from red/purple to blue of the suspension (Figure 6a). Thus, after the addition of 1 M NaCl, the maximum absorption wavelength (λ_max_) of enzyme/NPs suspensions was measured by UV-Visible spectroscopy depending on the mixture ratio, *R*/*R*_th_. Figure 6b,c shows the change in λ_max_ (%), i.e., the relative shift in wavelength/λ_max_(NP) at different mixture ratios. In the absence of enzymes (*R*/*R*_th_ = 0), NP7 and NP40 suspensions presented the higher shift in λ_max_, with 30% and 40% change corresponding, respectively, to 160 and 200 nm shift. The raw data are given in Figure S9 in the Supplementary Materials. On the contrary, NP11 suspensions appeared to be more stable with only a λ_max_ shift of 5% (24 nm). In the presence of GOx (160 kDa), the addition of salt to the NPs suspension induced a smaller shift of the λ_max_ with a threshold value *R*/*R*_th_ where no aggregation is observed for all studied NPs. In the case of NP7 and NP11, this threshold value is equal to 1 where a monolayer of GOx is formed on the surface of NPs. In comparison, the aggregation of NP40 is prevented for *R*/*R*_th_ = 2, suggesting the adsorption of more than one layer of the enzyme. A consequent change of GOx conformation could occur on large NPs leading to a larger footprint of the enzyme on a flat surface [33] than on small NPs, with a high curvature [22]. Upon adsorption, the enzymes could also unfold leading to the formation of two layers.

In the presence of HRP (44 kDa), the NPs aggregation is fully prevented for larger values of *R*/*R*_th_, i.e., 12 for NP7, and 9 for NP11. We can notice that partial prevention of the aggregation is observed for lower *R*/*R*_th_ values, indicating the effective adsorption of some HRP at the surface of NPs. In the case of NP40, the aggregation was observed for all the tested values of *R*/*R*_th_. Finally, the enzyme/NPs bioconjugates were observed at *R*/*R*_th_ = 10 by TEM after uranyl acetate negative staining (Figure 6d). In comparison to NP11, GOx/NP11 bioconjugates presented a light grey corona of 5 nm due to the attachment of the protein to the NPs as previously reported [23]. In the case of HRP/NP11, no light grey corona can be observed suggesting either a non-homogenous HRP coating or a thin unfolded HRP layer. Indeed, no close contact between the NPs was observed as for native NP11 (Figure 6d).

**Figure 7:**
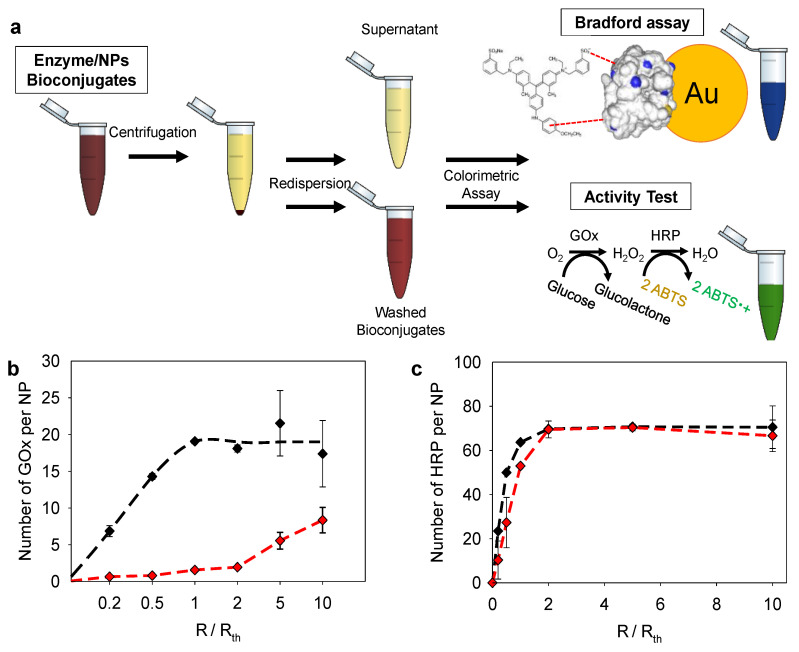
(**a**) Illustration of the Bradford and activity assays performed on enzyme/NP bioconjugates. Number of (**b**) GOx and (**c**) HRP per NP11 at different *R*/*R*_th_ mixture ratios, determined by Bradford (black curve) and activity assay (red curve), taking as a reference the activity of the enzyme-free in solution.

Further studies were then performed to evaluate the number of enzymes per NPs and their activity in comparison to the enzyme solution. These assays were performed for GOx/NP11 and HRP/NP11 which gave the best sensitivities for the electrodeposited coating. After the addition of the enzyme solution to NP11 suspension at a given *R* (enzyme/NPs molar ratio) for 30 min, the mixture was centrifuged to separate the enzyme/NPs bioconjugates from the unbound enzymes (Figure 7a). The number of enzymes per NP was determined by the Bradford test and the enzymatic activity using a classical colorimetric test. Figure 7b,c shows the results obtained in number of enzymes per NP with the enzymatic activity in the equivalent of the enzyme activity in solution.

For both enzymes, the number of enzymes per NPs increased with the enzyme/NPs (*R*/*R*_th_) molar ratio reaching a plateau of around 20 enzymes per NPs for GOx at *R*/*R*_th_ = 1, close to the theoretical monolayer ratio value. Regarding the enzymatic activity, the equivalent number of GOx continuously increased until 10 up to *R*/*R*_th_ = 10. It is interesting to cross-check this information with the final sensitivity of the biosensor obtained at the high *R*/*R*_th_ ratio (Figure 5a). Indeed, the free bioconjugates in solution keep a constant enzymatic activity at high ratio (R/R_th_ = 10), this corroborates the fact that at high ratio the sensitivity loss of the GOx/NPs coating does not come from the deposition of non-active enzymes but rather from the impossibility to electrodeposit these bioconjugates. At a low mixture ratio (*R*/*R*_th_ ≤ 2), mainly denaturated GOx adsorbed on the NP11 leading to a weak enzymatic activity (10% of adsorbed GOx are active). Upon adsorption, the enzymes are probably unfolded due to the strong interactions with TA, at the surface of NPs. Interacting strongly through hydrogen bonds and hydrophobic interactions, TA is known to inhibit the catalytic activity of enzymes by modification of their conformation or substrate deprivation [34,35]. At higher mixture ratio *R*/*R*_th_ = 10, almost 50% of adsorbed GOx is enzymatically active. More enzymes interact with the surface leading to fewer interactions with each enzyme and less unfolding. Knowing that GOx has an ellipsoidal shape (6.0 × 5.2 × 7.7 nm^3^), the adsorption of the enzyme could shift from the long side to the short side with the increase in the enzyme/NPs ratio [22]. In the case of HRP, the number of enzymes per NPs and the enzymatic activity increased with the mixture ratio, following the same behavior. The activity of HRP was comprised between 50 and 100%. The number of enzymes per NP reached a plateau at *R*/*R*_th_ = 2 with 100% of HRP active. The same trend was observed in the case of HRP/NPs coating (Figure 5b) with an increase of the enzymatic activity until *R*/*R*_th_ ~ 1–2 followed by a plateau. This confirms the adsorption of more than one layer of HRP on NP11.

**Figure 8:**
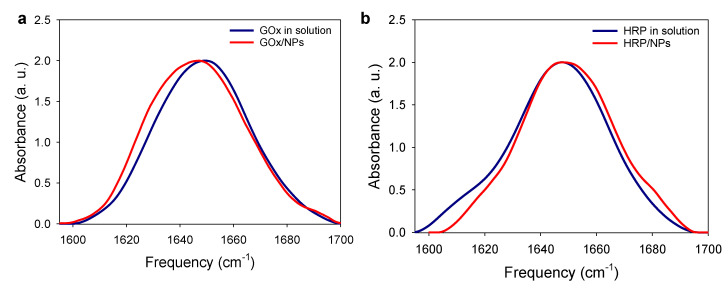
FTIR normalized spectra, amide I band region (**a**) of GOx solution (10 mg/mL) and GOx/NPs suspension and (**b**) of HRP solution (10 mg/mL) and HRP/NPs suspension, prepared in D_2_O.

To assess the secondary structure of the enzymes adsorbed on NPs, FTIR spectra of the GOx/NPs and HRP/NPs bioconjugates were recorded in deuterium oxide and compared to GOx and HRP in solution, respectively (Figure 8). Upon adsorption on NPs, the secondary structure of GOx is strongly modified with a shift of the maximum peak from 1650 to 1646 cm^−1^ and a peak broadening towards lower frequency. The loss in activity of GOx, through thermal denaturation, was shown to be linked to the loss of α-helix and increase in the unordered structure, i.e., a shift towards lower frequency [36]. On the contrary, adsorbed HRP enzymes have similar secondary structure to HRP in solution. The maximum peaks are at the same value and a narrower shape. Structural studies of HRP showed that the amount of α-helix are important for the activity of the enzyme [37]. Indeed, the inactivation of HRP is usually obtained by a strong decrease of alpha-helix structures [38]. Upon adsorption, the tertiary structure of HRP is probably modified without affecting drastically the secondary structure and thus its activity.

To summarize independently of the NPs size, the electro-cross-linked GOx/NPs coatings present an optimal value of the sensitivity towards glucose when *R*, the enzyme/NPs molar ratio, is close to the theoretical *R*_th_ of a GOx monolayer (*R*/*R*_th_ = 1). Despite the loss of enzymatic activity caused by conformational changes, high sensitivity of the GOx/NPs coating was obtained when a monolayer of GOx was formed on NPs. At high values of *R*/*R*_th_ above 1, adsorbed GOx on NPs had a better enzymatic activity but form a very dense monolayer preventing efficient electrodeposition of the coating leading to a decrease of the sensitivity. The gallol moieties on the surface of NPs are probably not accessible to obtain the cross-linking with other coated NPs. In the case of HRP/NPs coatings, the sensitivity increased linearly with *R*/*R*_th_ until a value close to 1–2 followed by almost a plateau for all tested NPs sizes. Contrary to GOx, unfolded HRP can be adsorbed on NPs until the quantity of two layers keeping its enzymatic activity. HRP layers onto NPs could not be observed by TEM suggesting the formation of a thin layer of HRP. In this case, the electrodeposition of HRP/NPs bioconjugates is efficient even at a high *R*/*R*_th_ mixture ratio. The gallol moieties of NPs seem to be accessible to obtain the cross-linking.

## 3. Conclusions

The sensitivity of electro-crosslinked GOx/NPs and HRP/NPs biosensors was optimized by adjusting the NPs size and the enzyme/NP molar ratio of the electrodeposition solution. Contrary to HRP/NPs biosensors, the NPs size has a strong effect on the sensitivity of GOx/NPs with an optimal size of 11 nm. In both cases, the increase in enzyme/NPs molar ratio leads to an increase in the sensitivity until a threshold close to the theoretical value of an enzyme monolayer. Above this threshold value, the sensitivity of the GOx/NPs coatings towards glucose decreased contrary to the HRP/NPs coatings that reached almost a plateau. A comprehensive evaluation of the colloidal state of the enzyme/NPs bioconjugates allows us to explain the main difference between the two enzymes. At high enzyme/NPs ratios, a too dense layer of GOx covers the surface of NPs preventing efficient electrodeposition of the coating leading to a decrease in its sensitivity. In the case of HRP, two layers of active enzymes can be adsorbed onto NPs. The HRP layer, not visible by TEM, seems to be thin and allowed access to gallol moieties ensuring efficient electrodeposition at a high enzyme/NPs ratio. This study opens the route toward the rational design of nanohybrid biosensors taking into account the NPs and the enzyme sizes.

## 4. Materials and Methods

### 4.1. Chemicals

Tannic acid (TA, M_w_ = 1701.23 g·mol^−1^, CAS 1401-55-4), ferroceneboronic acid ≥ 95% (FcB(OH)_2_, M_w_ = 229.85 g·mol^−1^, CAS 12152-94-2), glucose oxidase from *Aspergillus Niger* sp. Type X-S (GOx, ~168 U·mg^−1^, M_w_ = 160 kDa, G7141, CAS 9001-37-0), horseradish peroxidase (HRP, ~250 U·mg^−1^, M_w_ = 44 kDa, CAS 9003-99-0), hydrogen peroxide solution 30 wt.% (H_2_O_2_, M_w_ = 34.01 g·mol^−1^, CAS 7722-84-1), Bradford reagent (reference B6916), and phosphate buffered saline tablets were purchased from Sigma-Aldrich (St. Louis, MO, USA). Ferrocene methanol (FcOH ≥ 95%, Mw = 216.1 g·mol^−1^, CAS 1273-86-5) was retrieved from FluoroChem (Hadfield, UK). Hydrogen tetrachloroaurate(III) hydrate (HAuCl_4_, M_w_ = 393.83 g·mol^−1^, CAS 27988-77-8), d-(+)-glucose anhydrous 99% (M_w_ = 180.16 g·mol^−1^, CAS 50-99-7), and 2,2′-azino-bis(3-ethylbenzothiazoline-6-sulfonic acid) (ABTS 98 %, M_w_ = 548.68 g·mol^−1^, CAS 30931-67-0) were purchased from Alfa Aesar (Haverhill, MA, USA). Sodium chloride ≥99.5% (NaCl, M_w_ = 58.44 g·mol^−1^, CAS 7631-99-4), and potassium carbonate ≥99.5% (K_2_CO_3_, M_w_) =138.21 g·mol^−1^, CAS 209-529-3) were purchased from Fischer (Pittsburgh, PA). All chemicals were used as received and solution were prepared using Milli-Q water with 18.2 MΩ·cm resistivity.

### 4.2. Tannic Acid Capped Gold Nanoparticles Synthesis (NPs)

0.3 mM TA and 2 mM HAuCl_4_ solutions were prepared in Milli-Q water. In the case of NP7, the TA solution was prepared with 0.1 mM FcB(OH)_2_. This reducer was found to enhance a better stabilization effect of the NPs compared to FcOH thanks to boronate ester formation. In all cases, the TA solution was adjusted at pH 7 with 150 mM K_2_CO_3_. The AuCl_4_^−^ solution was not adjusted for NP11 (pH ~ 2.6) and adjusted at pH 10 for NP40 with 150 mM K_2_CO_3_. The solutions were left to equilibrate for 30 min before the final pH measurement. The TA and AuCl_4_^−^ solutions were filtered on 0.45 µm regenerated cellulose (RC) filters and flushed with argon for 5 min. For the gold NPs synthesis, 8 mL of AuCl_4_^−^ solution was slowly added to 12 mL of TA solution with a peristaltic pump at 500 µL·min^−1^ (ISM596, Ismatec, Switzerland) under 750 rpm agitation. During the addition, 20 µL of 150 mM K_2_CO_3_ was added every 2 min to compensate the acidification. A dark reddish solution was obtained with a final TA/Au ratio of 0.22. The obtained TA capped gold nanoparticles were further flushed with argon and stored for up to 2 months at 4 °C to prevent TA oxidation. Before use, the synthesized NPs solution was centrifuged for a different amount of time (Table S2 in the Supplementary Materials) at 13,500 rpm (12,200× *g*) with a VWR Microstar 12 set-up to remove all unbound oxidized polyphenols from the solution and FTI further redispersed in water or enzymes/FcOH mixtures.

### 4.3. NP Characterizations

The size distribution of NPs was determined from TEM micrographs using ImageJ on 30 NPs dimensions along the *x* and *y*-axis to obtain the mean and standard deviation on 60 measurements. The NPs concentration was accurately determined using two different methods (Table S1 in the Supplementary Materials). The first one was based on the absorbance value at the plasmon resonance wavelength (524, 528, and 544 nm for 7, 11, and 40 nm diameter NPs, respectively) and the extinction coefficient of NPs [39]. The second method was based on the calculation of the theoretical concentration assuming a homogeneous distribution of gold in all NPs (Equation (1)). Both methods gave similar results with a difference of less than 10%. A_λmax_ is the absorbance of washed undiluted NPs solution at the maximum absorbance wavelength, l the optical length path, ε is the molar absorptivity at 532 nm, N_A_ the Avogadro number, M_w_(Au), the molar mass of gold (196.96 g·mol^−1^), c(Au) = 0.8 mM the final concentration of gold in solution, d(Au) the density of gold (19.32 g·cm^−3^) and D_NPs_ the diameter of NPs.

(1) $$c\;\left(NPs\cdot L-1\right)=\frac{A_{\lambda max}\times N_{A}}{\varepsilon \times l}=\frac{c\left(Au\right)\times M_{w}\left(Au\right)}{d\left(Au\right)\times \frac{4}{3}\pi {\left[\frac{D_{NPs}}{2}\times 10^{-7}\right]}^{3}}$$

### 4.4. Dynamic Light Scattering Characterization

The hydrodynamic size was recorded with a 4-fold diluted NPs solution using DTS1070 cell and a Nanosizer (Malvern Panalytical, UK) at a collection angle of 173° and a 633 nm monochromatic laser. Measurements were following the standard operating procedure (EUNCL-PCC-001) from the European Nanomedicine Characterization Laboratory. A viscosity of 0.89 mPa·s and a refractive index of 1.332 was used for the water. A resting time of 5 min was set to enable temperature equilibrium at 25 °C. Five measurements with 12 runs of ten seconds each were performed and averaged. A Laplace inversion PSD analysis was used to fit the autocorrelation function assuming multiple diffusion coefficients may be presents in the sample.

### 4.5. Enzyme Characterization

The enzyme concentration was determined using two different spectroscopic methods. The Beer-Lambert law was applied using the absorbance value at 280 nm and the extinction coefficient of 263 mM^−1^·cm^−1^ for GOx and 19.6 mM^−1^·cm^−1^ for HRP. The Bradford assay was performed using 5 µL of enzyme prepared at 1 mg·mL^−1^ mixed for 30 min with 250 µL of Bradford reagent according to the supplied technical note. The absorbance value was measured at 595 nm and compared to a calibration curve, obtained using bovine serum albumin. The concentration of the commercial enzymes batches was 0.76 ± 0.05 mg/weighted mg for GOx and 0.90 ± 0.01 mg/weighted mg for HRP. Both methods gave similar results with a deviation lower than 4%.

### 4.6. Enzyme/NPs Electrodeposition Solution

5 mM FcOH solution was prepared in water using an ultrasonic bath for 30 min to quicken its dissolution with a temperature kept below 40 °C. After cooling at room temperature, the solution was filtered with a 0.2 µm RC syringe filter. The enzyme (GOx or HRP) was dissolved in the FcOH solution at the targeted concentration by taking into account the enzyme commercial purity. 300 µL of enzyme/FcOH solution was further added to a 10-fold pre-concentrated NPs solution (previously centrifuged and washed). After 30 min, 15 µL of 1 M NaCl solution was added to reach 50 mM NaCl in the FcOH/enzyme/NPs suspension. For the electrodeposition of NPs, a similar procedure was followed without the addition of enzyme in the FcOH solution.

### 4.7. Electrochemical Quartz Crystal Microbalance (EC–QCM)

Q-Sense E1 from Q-Sense AB (Sweden) was used to perform the electrochemical quartz microbalance (EC–QCM) experiments by monitoring the changes in the resonance frequency *f_ν_* and the dissipation factor *D_ν_* of an oscillating gold-coated quartz crystal (QCM5140TiAu120-050-Q, QuartzPro, Sweden) upon adsorption of a viscoelastic layer (ν, the overtone number, equal to 1, 3, 5 and 7). Only the evolution of the third overtone at 15 MHz (*f*_3_) is presented. Electrochemical measurements were performed on a CHI660E apparatus from CH instrument (Austin, TX, USA) coupled with the Q-Sense E1 apparatus with a three-electrode system: The gold-coated QCM sensor acted as the working electrode. A platinum electrode (counter electrode) on the top wall of the chamber and a no-leak Ag/AgCl reference electrode fixed in the outlet flow channel were used, respectively, as counter and reference electrodes. The internal volume of the EC–QCM cell was 100 µL and the electrode distances were fixed at 0.8 mm for working electrode/counter electrode) and 5 mm for working electrode/reference electrode. Before any experiments, the crystals were cleaned for 30 min using a UV/Ozone cleaner (ProCleaner, BioForce Nanoscience, Ames, IA, USA). Before every deposition, RE potential was carefully checked against FcOH/Fc^+^OH and the stabilization of all the frequencies was achieved. The deposition was initiated 10 min after injection (resting time) by applying a constant +0.7 V bias for 60 min (electrodeposition time). 

### 4.8. Determination of the Electrodeposition Efficiency

The Sauerbrey equation (2) was used to convert the frequency shift, measured by EC-QCM, into a deposited mass in ng·cm^−2^. The number of deposited NPs $\left(n_{d}\right)$ can be estimated knowing the geometric surface of the working electrode (the gold-coated QCM crystal) in contact with the electrodeposition solution (0.80 cm^2^ with a surface roughness <1 nm [29]) and the mass of a single NP (3). The overall number of NPs $\left(n_{c}\right)$ in the electrodeposition solution can be roughly estimated by the internal cell volume (100 µL) [29]. The ratio of the two values, $n_{d}$ and $n_{c}$, gives the efficiency of the electrodeposition process (4). In the following equations, C is a constant of 17.67 ng·cm^−2^·Hz^−1^ (5 MHz AT-cut quartz crystal), $\frac{\Delta f_{n}}{n}$ is the value in the frequency shift at the *n* overtone, $R_{NPs}$ is the radius of the nanoparticle, $M_{w,Au}$ = 19.32 g·cm^−3^ is the molar mass of gold, n_d_ is the number of electrodeposited NPs and $n_{c}$ is the number of colloidal NPs present in the electrodeposition solution in contact with the working electrode (gold-coated QCM crystal).

(2) $$\mathrm{Sauerbrey}\;\mathrm{equation}:\;\Delta m=-C\times \frac{\Delta f_{n}}{n}$$

(3) $$\mathrm{Mass}\;\mathrm{of}\;a\;\sin\mathrm{gle}\;\mathrm{NP}:\;m\;\left(g\right)=\frac{4}{3}\;\pi \times {\left(10^{-7}\times R_{\mathrm{NPs}}\right)}^{3}\times M_{w,\mathrm{Au}}$$

(4) $$\mathrm{Electrodeposition}\;\mathrm{efficiency}:\;e.e\;\left(\%\right)=\frac{n_{d}}{n_{c}}\times 100$$

### 4.9. Electroactive Surface Area (EASA) Determination

To evaluate the EASA, the following test was performed before and after the deposition of NPs coatings. Cyclic voltammograms were registered at various scan rates from 5 to 500 mV·s^−1^ on the bare gold crystal (working electrode) in contact with 0.5 mM FcOH in 50 mM NaCl solution. After the electrodeposition, the NPs coatings were rinsed with 50 mM NaCl for 10 min to remove all the free FcOH until only the capacitive signal was measured. The same CV cycles were then performed on the electrodeposited NPs coating

The Randles-Sevcik Equation (5) was then used to estimate the EASA.

Randles-Ševčik:

(5) $$i_{p}=0.446nFAC^{0}{\left(\frac{nFvD_{0}}{RT}\right)}^{1/2}\{\begin{array}{c} i_{p}:current\;peak\;(A)\\ n:number\;of\;exchanged\;electron\\ A:electroactive\;surface\;({\mathrm{cm}}^{2})\\ v:scan\;rate\;(V\cdot s^{-1})\\ D_{0}:diffusion\;coefficient\;({\mathrm{cm}}^{2}\cdot s^{-1})\\ C_{0}:ferrocene\;concentration\;(\mathrm{mol}\cdot {\mathrm{cm}}^{-3}) \end{array}$$

### 4.10. Scanning Electron Microscopy (SEM)

After a washing step with water and a drying step, the morphology of the electrodeposited coatings was characterized by a Hitachi SU 8010 Ultra High-Resolution Field Emission Scanning Electron Microscope (Tokyo, Japan). Secondary electron micrographs were retrieved with an accelerating voltage of 1 kV, an emission current of 10 µA and a working distance of 3 mm.

### 4.11. Electrochemical Determination of the Biosensor Sensitivity

The same electrode set-up used during the electrodeposition was used to perform the electrochemical biosensor performance with the gold-coated QCM crystal as the working electrode. All the electrochemical measurements were carried out in 10 mM PBS solution on the CHI 660E electrochemical workstation (CH Instruments, Austin, TX, USA). Cyclic voltammetry measurements were performed by injecting 600 µL of glucose solutions from 50 µM to 50 mM at 1.4 mL·min^−1^ and in the presence of FcOH. The chronoamperometric curves were performed at a constant potential of 0.25 V (vs. Ag/AgCl) in the presence of 0.5 mM FcOH for glucose, and of 0.19 V (vs. Ag/AgCl) in the presence of 0.1 mM FcOH for H_2_O_2,_ unless otherwise stated. The FcOH concentration was optimized to acquire the upper limit of the biosensor linearity range slightly above the upper limit of the substrate physiological range in blood (~10 mM for glucose and 100 µM for H_2_O_2_) [40,41,42]. The sensitivity plots were settled using the value of the current at the steady-state after the overshooting due to the injection of the analyte. A surface area of 0.8 cm^2^, corresponding to the exposed area of the gold QCM sensor, was used for the current density calculation [43].

### 4.12. Enzymes/NPs Bioconjugate Observation with Transmission Electron Microscopy (TEM)

5 μL of the bioconjugate suspension was deposited onto a freshly glow discharged carbon-covered grid (400 mesh). The suspension was left for 2 min and then the grid was negatively stained with 5 μL of uranyl acetate (2% in water) for another 1 min and finally dried using a filter paper. The grids were observed at 200 kV with a Technai G2 (FEI) microscope. Images were acquired with an Eagle 2k (FEI) ssCCD camera and further analyzed with ImageJ.

### 4.13. Flocculation Test of the Enzyme/NPs Bioconjugates

100 µL of enzyme solution was mixed with 100 µL of NPs suspension varying their concentrations to acquire different *R*/*R*_th_ ratios from 0 to 15. After a resting time of 30 min, the flocculation test was performed by adding 50 µL of 5 M NaCl solution to the mixture to reach a final concentration of 1 M NaCl. The absorbance spectra of the different enzyme/NPs suspensions were recorded with a spectrophotometer (SAFAS Xenius, Monaco City, Monaco). The shift in the value of the maximal absorbance (λ_max_) allowed characterizing the aggregation of the enzyme/NPs bioconjugates thanks to the color change (Figure 6a).

### 4.14. Bradford Assay Enzyme/NPs Bioconjugates

The Bradford assay allowed determining the quantity of enzyme adsorbed on the surface of NPs. It was performed in the linear range with the initial concentrations adjusted to remain below 0.74 and 0.94 in absorbance units for HRP and GOx, respectively. The enzyme solution and NPs suspension were mixed at different *R*/*R*_th_ ratios. After a resting time of 30 min, the mixture solutions were centrifuged to remove the unbound enzymes using the same centrifugation time as used for NPs suspensions (Table S2 in the Supplementary Materials). 100 µL of the enzyme/NPs suspension was mixed with 200 µL of the Bradford reagent for 30 min. The absorbance value was measured at 595 nm and the background subtraction was performed using NPs suspension as a blank since TA molecules react with the coomassie blue to a lesser extent.

### 4.15. Colorimetric Enzymatic Tests for Bioconjugates

The enzyme/NPs bioconjugates were prepared similarly to the one for the Bradford assay and centrifuged to remove unbound enzymes. Colorimetric tests were performed to evaluate the enzymatic activity either at constant NPs or enzyme concentrations. All the tests were performed by adding the enzyme substrate in PBS 10 mM pH 7.4 at room temperature to mimic the operating condition in the physiological medium. The supernatant was also tested to ensure that the uncertainty of the remaining ~2% vol supernatant was minimal compared to bound enzymes. The total amount of NP from the supernatant and the washed bioconjugates was equal to the activity of the initial enzyme/NPs mixture (±10%) independently of the initial *R*/*R*_th_ ratio. This confirms that the accuracy of the colorimetric tests. In the case of HRP, the assay consists in mixing 10 µL of HRP/NPs bioconjugates with 40 µL water followed after one hour by 150 µL of 10 mM of chromogenic substrate/10 mM H_2_O_2_ in 10 mM PBS. In the case of GOx, the assay was based on a dual enzymes system using HRP and the chromogenic substrate 2,2′-azino-bis(3-ethylbenzothiazoline-6-sulfonic acid) (ABTS) both in large excess in regards to GOx concentration. 10 µL of GOx/NP bioconjugates were prepared with 40 µL of NPs at their initial concentration (value indicated in Table S1 in the Supplementary Materials). After an incubating time of 1 h, 150 µL of 10 mM PBS solution containing 1 mM ABTS and 10 mM glucose was added to reach ~50 µg·mL^−1^. The activity of the enzyme bound to NPs was determined after a centrifugation procedure (13,500 rpm, 60 min) and a redispersion in an equivalent volume of water. The absorbance of the coloured ABTS^•+^ was recorded at 420 nm every 15 s (ε_420nm_ = 36 mM^−1^ cm^−1^) and the slope of this curve enables to determine the kinetic of glucose disappearance proportional to the enzyme activity at substrate saturation (6). This slope was calculated on the first ten minutes of the linear range and similar result at a given *R*/*R*_th_ ratio was obtained with variation of either enzyme concentration or nanoparticle concentration. From a calibration curve made from the dilution of the enzyme only in solution, a change of 1 absorbance unit per minute was found to be equivalent to 0.4295 µmoles·L^−1^ of active enzyme (R^2^ = 0.999). Equation (7) was further used to determine the equivalent number of active enzymes per nanoparticles for each *R*/*R*_th_ ratio.

(6) $$\mathrm{Glucose}+O_{2}\;\overset{GOx}{\to }\;\mathrm{glucolactone}+H_{2}O_{2}$$

(7) $$H_{2}O_{2}+\mathrm{ABTS}\;\overset{HRP}{\to }\;H_{2}O+{\mathrm{ABTS}}^{•+}\;\mathrm{Number}\;\mathrm{of}\;\mathrm{active}\;\mathrm{enzymes}\;\mathrm{per}\;\mathrm{NP}=\frac{{\mathrm{slope}\times 10}^{-6}{\times N}_{A}}{0{.4295\times c}_{\mathrm{NPs}}}$$

### 4.16. Enzyme Conformation onto NP from FTIR Experiments

The FTIR spectra of GOx, HRP, GOx/NPs, HRP/NPs were recorded in D_2_O solution using a Vertex 70 spectrometer (Bruker, Germany) using a DTGS detector. Spectra were recorded in the Attenuated Total Reflection (ATR) mode using single reflection diamond ATR by averaging 28 interferograms between 800 and 4000 cm^−1^ at 2 cm^−1^ resolution, using Blackman-Harris three-term apodization and Bruker OPUS/IR software (v7.5). Data processing was performed using OPUS 7.5 software (Copyright Bruker Optik GmbH 2014). The IR spectrum was smoothed using a 25-point smoothing function and cut between 1560 and 1720 cm^−1^. After adjustment of the baseline, the spectrum was normalized using a normalization “min-max” method.

## Data Availability

All the data supporting can be found in the text and the supporting of this paper.

## Supplementary Materials

The following supporting information can be downloaded at: https://www.mdpi.com/article/10.3390/molecules27103309/s1. Table S1: GOx and HRP size obtained from DLS and TEM analysis; Table S2: Description of the NPs used in this study; Table S3: Calculated Rth, the theoretical enzyme/NPs molar ratio; Figure S1: Hydrodynamic size of the tannic acid capped gold nanoparticles measured by DLS; Figure S2: SEM micrographs of a bare gold QCM; Figure S3: Illustration of enzyme footprint and the estimation of the surface coverage; Figure S4: Cyclic voltammogram of HRP/NP11 and GOx/NP11 film in presence of enzyme substrate and evolution of the deposited mass and sensitivity with the electrodeposition time. Figure S5: Sensitivity plot of GOx/NP11 and HRP/NP11 obtained by variation of *R*/*R*_th_ ratio; Figure S6: Evolution of the deposited mass obtained by variation of *R*/*R*_th_ ratio. Figure S7: Chronoamperogram in the presence of H2O2 for NP11 and HRP controls; Figure S8: Senstitivity plot of HRP/NP11 as a function of *R*/*R*_th_ ratio and FcOH concentration; Figure S9: Evolution of the maximum wavelength (λmax) of NPs as a function of the mixture ratio.
