# Supplementary material for: Optimization of Nanohybrid Biosensors Based on Electro-Crosslinked Tannic Acid Capped Nanoparticles/Enzyme
Source: Molecules. 2022 May 21;27(10):3309. doi: 10.3390/molecules27103309 (PMC9144895; doi:10.3390/molecules27103309)
Supplement: Supplementary file 1 [file molecules-27-03309-s001.zip › molecules-1680341-supplementary.pdf]

Supplementary information

Optimization of Nanohybrid Biosensors Based on Electro-crosslinked Tannic Acid Capped Nanoparticles/Enzyme

Rémy Savin [1], Christian Blanck[1], Nour-Ouda Benzaamia [1] and Fouzia Boulmedais [1,2,] *

[1] Université de Strasbourg, CNRS, Institut Charles Sadron UPR 22, 67034 Strasbourg, France.

[2] International Center for Frontier Research in Chemistry, 67083 Strasbourg, France.

* Author to whom correspondence should be addressed.

Table S1: GOx and HRP size obtained from dynamic light scattering (DLS) and TEM analysis and comparison with the crystal structure size obtained from Wang et. al. 2019 and Cans et. al. 2007.

Size (nm)	DLS	TEM	PDB X-ray model
GOx (Sigma G7141)	6.9 ±0.5	7.3 ±0.7	1CF3[1] $6.0\times5.2\times7.7$ nm^3
HRP (Serva 31941.03)	5.5 ±0.5	4.3 ±0.7	1HCH[2] $6.0\times4.4\times4.0$ nm^3

Table S2: Description of the NPs used in this study

Sample name	Mean size by DLS (nm)	PDI	Centrifugation Time (min)	Concentration ($\times10^{15}$ NPs L^{-1})	Equivalent surface ($\times10^{18}$ eq nm^2 L^{-1})
NP7	9 ±2	0.33	120	45 ±4	~ 7.1 ±0.9
NP11	12 ±2	0.20	60	10 ±1	~ 4.4 ±0.7
NP40	38 ±7	0.54	15	0.2 ±0.1	~ 1.5 ±0.4

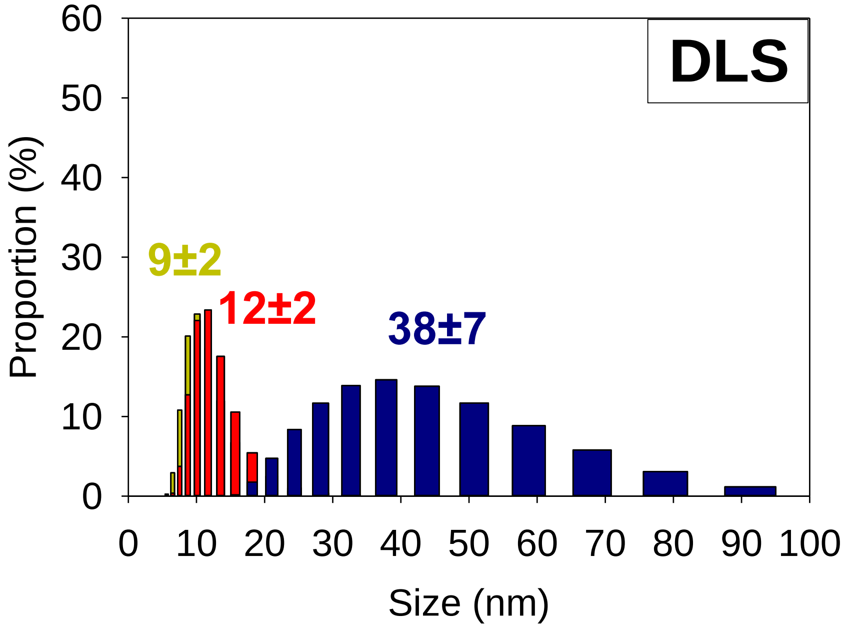

Figure S1: Hydrodynamic size of the tannic acid capped gold nanoparticles measured by dynamic light scattering, NP7 (yellow), NP11 (red) and NP40 (blue).

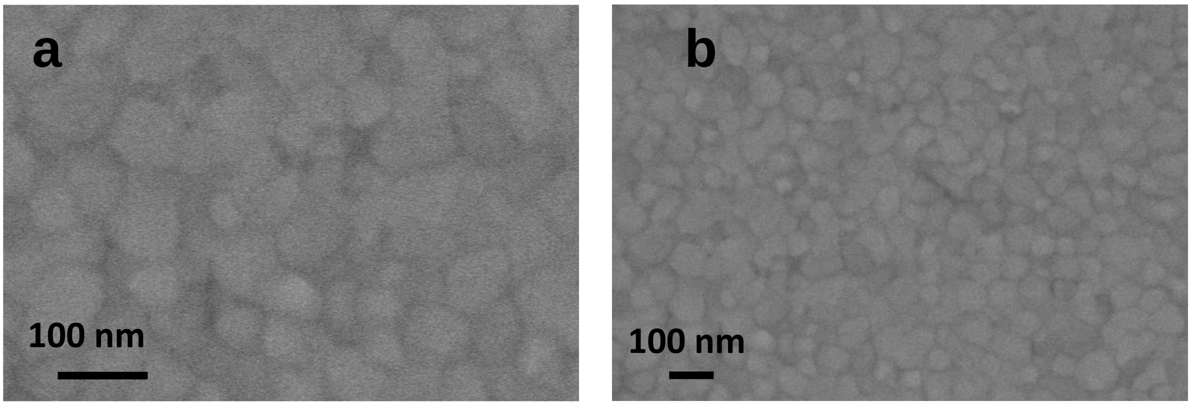

Figure S2: SEM micrographs of a bare gold QCM crystal used as working electrode for NPs at (a) high and (b) low magnification.

Section 1. Calculation of the molar ratio of enzyme/NPs to obtain an adsorbed monolayer (R_{th}). R_{th} was defined as the number of small non deformable enzyme spheres that can be stacked around a single nanoparticle. The theoretical ratio was defined as follows with R_{NP} the radius of NP and R_E the radius of the enzyme.

$$R_{th} = \frac{4 \times (R_{NP} + R_E)^2}{(R_E)^2}$$

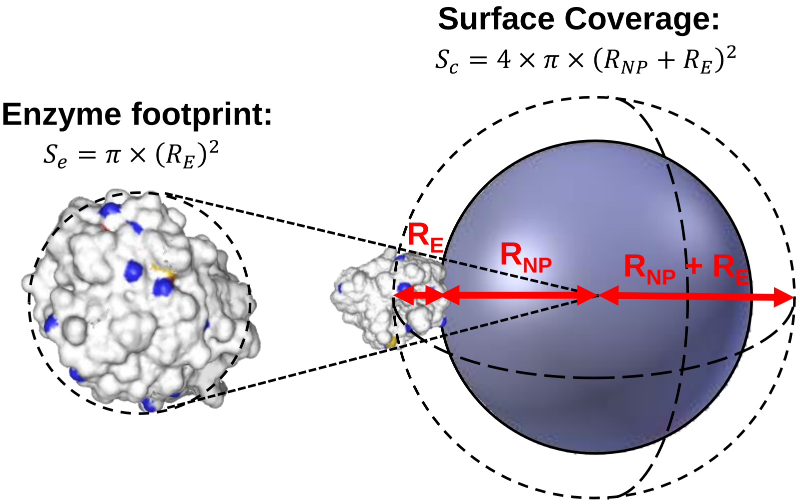

Figure S3: Illustration of enzyme footprint and the estimation of the surface coverage.

Even if this simple calculation of R_{th} is imperfect (enzymes are assumed to be an undeformable solid sphere and no compacity factor is taken into account), this formular gave a good approximation for the considered enzyme and NP size range. We compute an incremental algorithm (known as the Thomson problem) and then verify that the distance between all points

representing an enzyme is lower than $2\times R_{Enz}$ with Wolfram Mathematica. The solution found for GOx/NP10 with this algorithm is concordant with our simple estimation of R_{th} (Table S3).

Table S3: Calculated R_{th}, the theoretical enzyme/NPs molar ratio required to obtain an enzyme monolayer and an enzyme bilayer at the surface of NPs.

Sample name	NP size by DLS (nm)	GOx		HRP	
		R_{th}	bilayer	R_{th}	bilayer
NP7	7 ±1	16	79	20	92
NP11	11 ±2	27	111	36	136
NP40	42 ±6	201	531	298	751

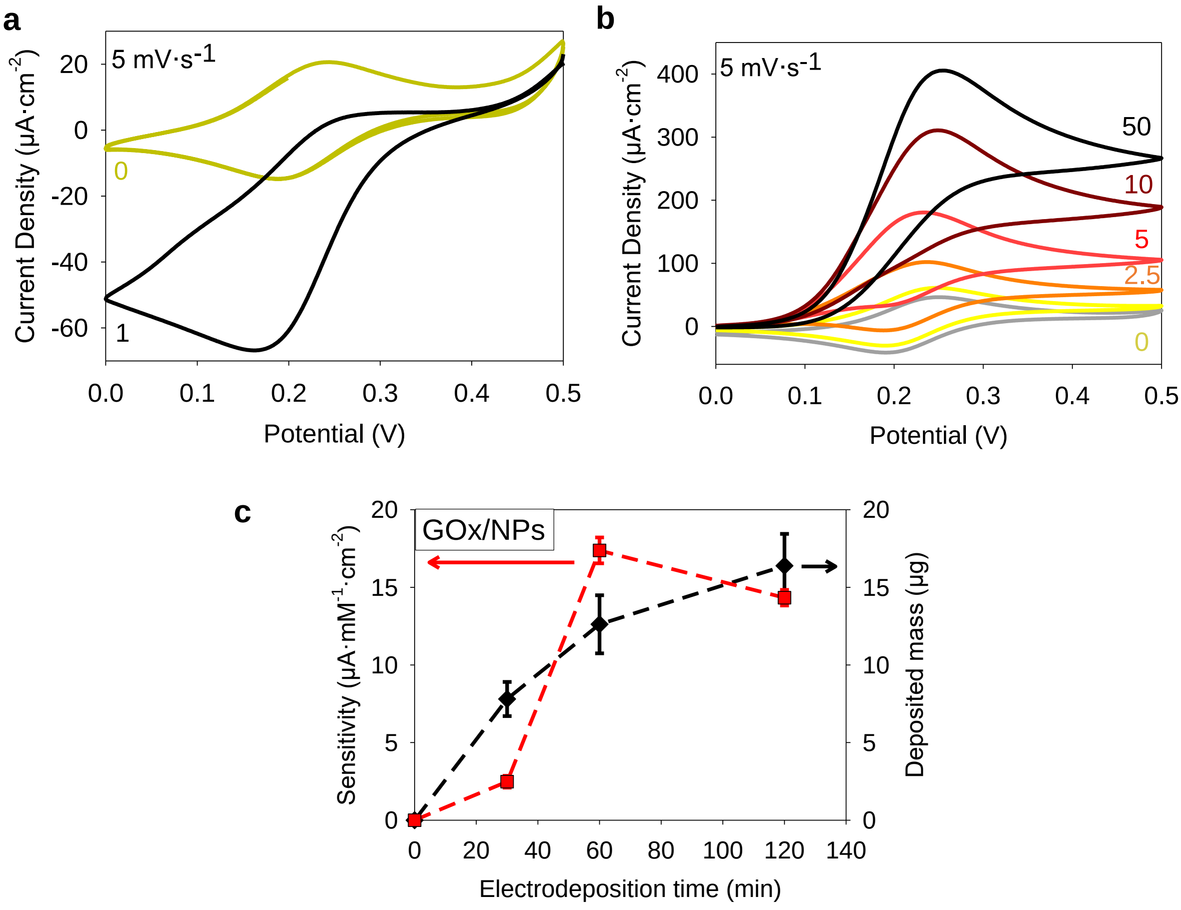

Figure S4: (a) Cyclic voltammogram at 5 mV s^{-1} of HRP/NP11 film built at $R/R_{th} = 1$ in the absence (yellow) and in the presence of 1 mM H_2O_2. H_2O_2 was dissolved in 100 µM FcOH / PBS 10 mM pH 7 solution. (b) Cyclic voltammogram at 5 mV s^{-1} of GOx/NP11 film built at $R/R_{th} = 1$ in the absence (yellow) and in the presence of different concentration of glucose from 1 to 50 mM. Glucose was dissolved in 500 µM FcOH / PBS 10 mM pH 7 solution. (c) Evolution of the deposited mass (black symbol) and sensitivity (red symbol) of GOx@AuNPs with the electrodeposition time performed at +0.7 V.

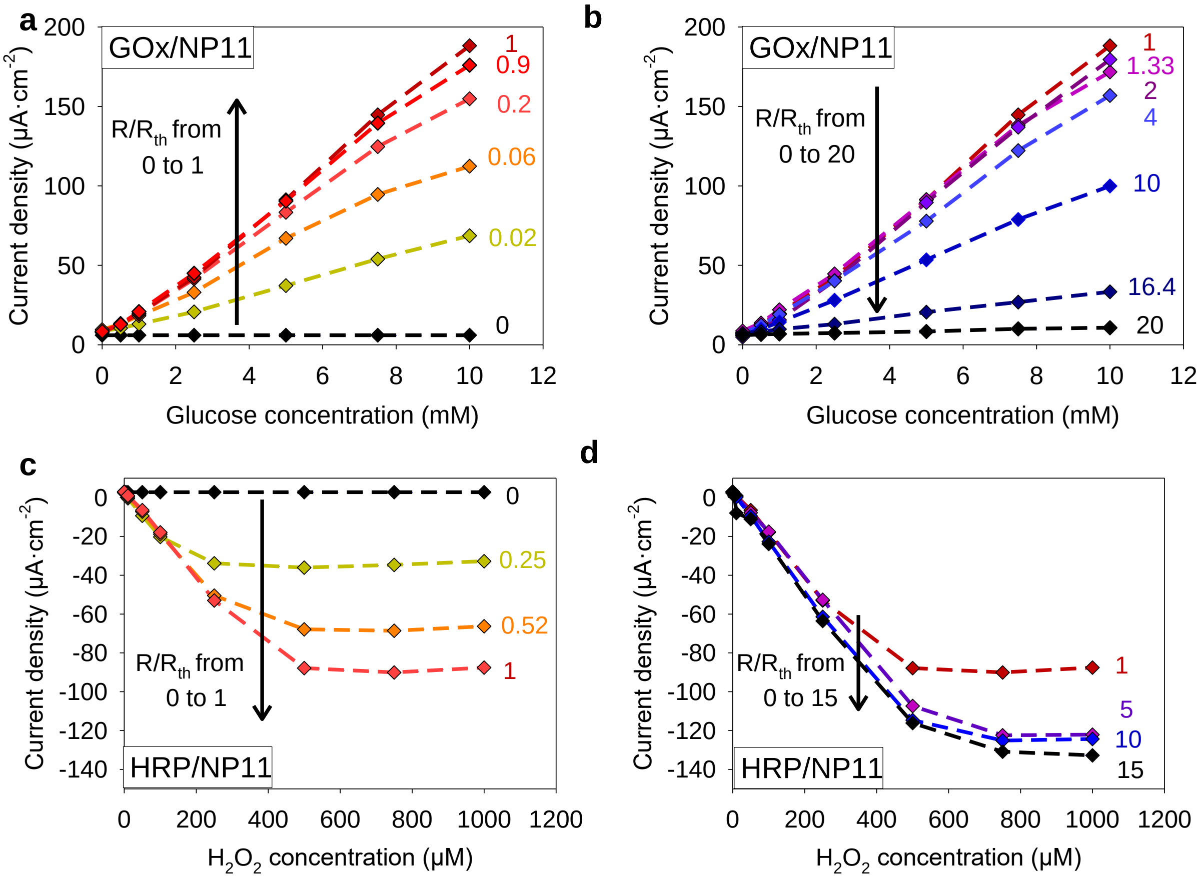

Figure S5: Sensitivity plot of (a,b) GOx/NP11 and (c,d) HRP/NP11 obtained by variation of the initial R/R_{th} ratio. (a,c) are the R/R_{th} values lower than 1 and (b,d) those higher than 1.

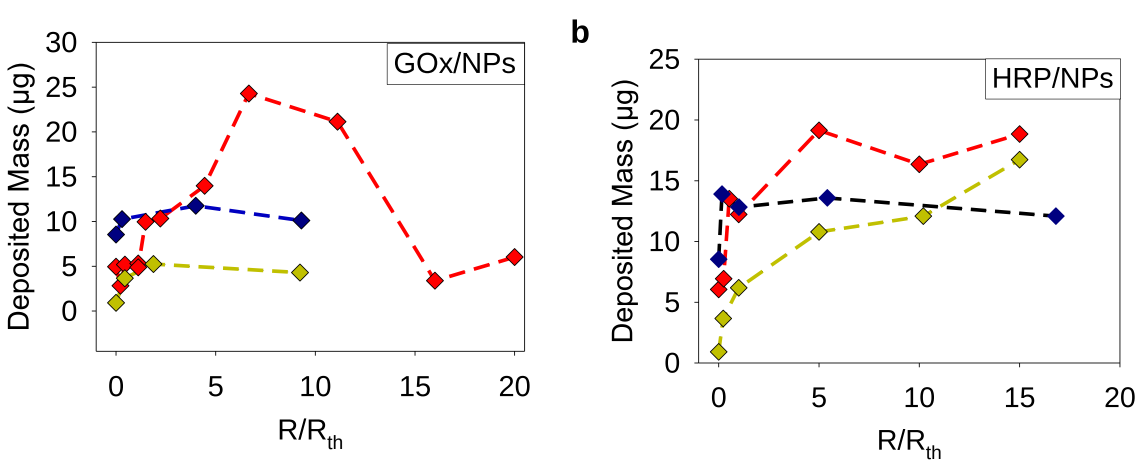

Figure S6: Evolution of the deposited mass, calculated from EC–QCM, using a geometric surface of 0.8 cm^2; (a) for GOx/NPs and (b) HRP/NPs coatings, prepared with NP7 (yellow square), NP11 (red diamonds), and NP40 (blue diamonds), as a function of the *R/R_{th}* mixture ratio (dashed line as a guide to the eye). *R* is the enzyme/NPs ratio of the electrodeposition solution and R_{th} is the theoretical enzyme/NPs molar ratio to obtain an enzyme monolayer at the surface of NPs.

Section 2. Different controls were carried out to validate the nature of the electrocatalytic signal observed in chronoamperometry. The sensitivity of NP11 film towards different H_2O_2 concentrations was studied. No electrocatalytic signal was obtained at 0.19 V for this type of film (Figure S7a). On the contrary the natural reduction of H_2O_2 directly at the electrode is observed at a lower potential such as –0.5 V. In the case of the homogeneous HRP/H_2O_2/FcOH system (Figure S7b), an electrocatalytic signal is observed at 0.19 V only in the presence of HRP, and this signal is dependent on the enzyme concentration. This electrocatalytic signal, only linked to the presence of HRP and not to the presence of NP11 alone, is also proven by cyclic voltammetry (Figure S8a) with the total disappearance of the ferrocene oxidation peak at 0.25 V and the overexpression of the reduction peak at 0.19 V.

H_2O_2/HRP/FcOH system:

$$2Fc^{+}OH \xrightarrow{\text{Reduction}} 2FcOH + 2e^{-} \text{(at electrode)}$$

$$H_2O_2 + HRP(Red) \xrightarrow{\text{Catalytic reduction}} H_2O + HRP(Ox)$$

$$HRP(Ox) + 2FcOH \xrightarrow{\text{Homogeneous Redox}} HRP(Red) + 2Fc^{+}OH$$

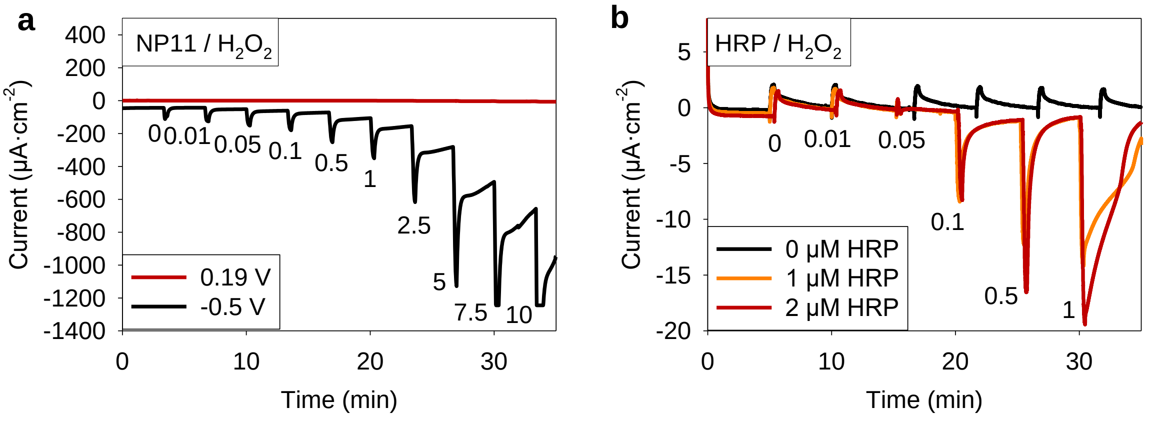

Figure S7: (a) Chronoamperogram in the presence of H_2O_2 at 0.19 and –0.5 V on a NP11 film prepared from NP11/FcOH solution. (b) Chronoamperogram at 0.19 V in presence of H_2O_2 and HRP at various concentration on a clean gold crystal. H_2O_2 was dissolved in 100 µM FcOH / PBS 10 mM pH 7 solution.

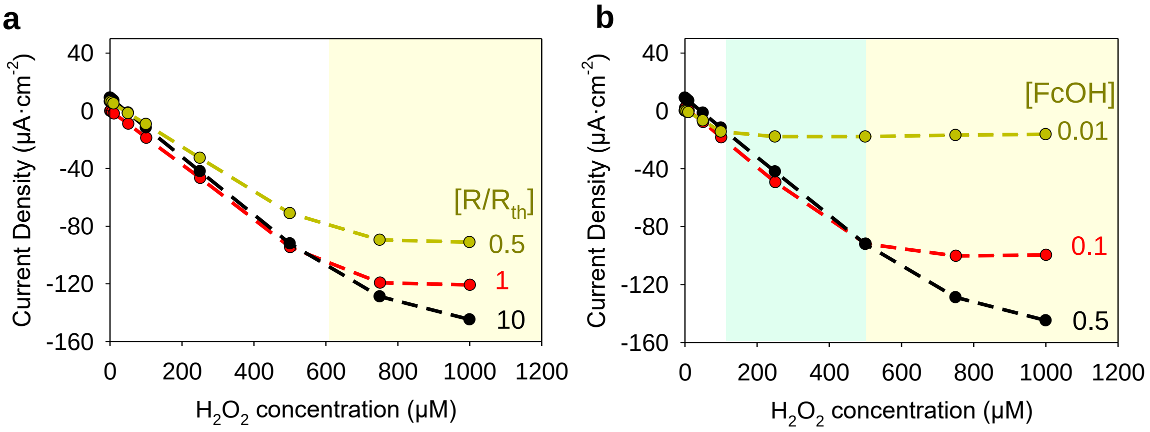

Figure S8: Current densities as function of H_2O_2 concentration measured for HRP/NP11 coatings: (a) built at different R/R_{th} mixture ratio (0.5, 1, and 10) using 0.5 mM FcOH as mediator and (b) built at R/R_{th} = 10 using 0.01, 0.1 and 0.5 mM FcOH as mediator.

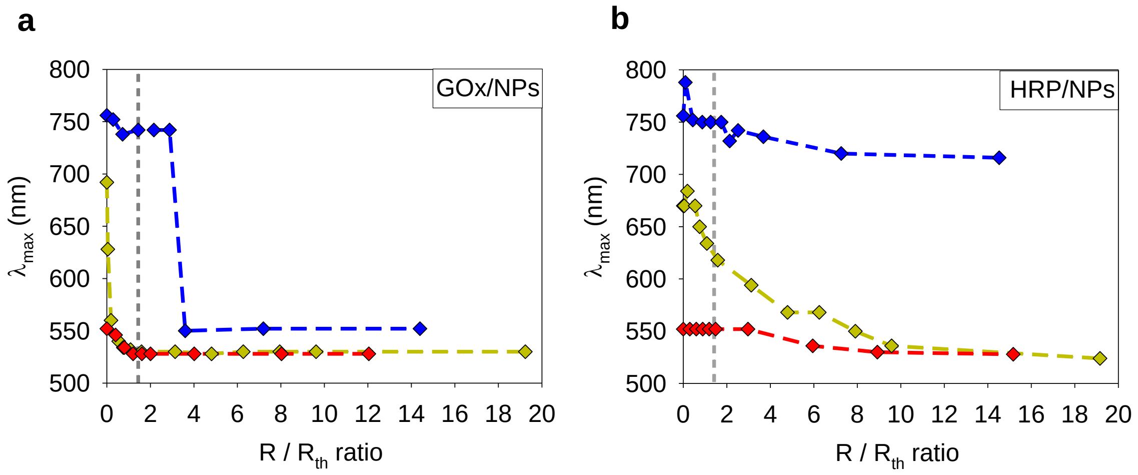

Figure S9: Evolution of the maximum wavelength (λ_{max}) as a function of the mixture ratio, R/R_{th} for (a) GOx/NPs and (b) HRP/NPs suspensions with NP7 (yellow), NP11 (red), and NP40 (blue).

References

(1) Wang, Y.; Jonkute, R.; Lindmark, H.; Keighron, J. D.; Cans, A.-S. Molecular Crowding and a Minimal Footprint at a Gold Nanoparticle Support Stabilize Glucose Oxidase and Boost Its Activity. *Langmuir* **2019**, *36*, 37–46.

(2) Cans, A. S.; Dean, S. L.; Reyes, F. E.; Keating, C. D. Synthesis and Characterization of Enzyme-Au Bioconjugates: HRP and Fluorescein-Labeled HRP. *NanoBiotechnology* **2007**, *3*, 12–22.
